# Supplementary material for: Are carbon emissions trading and green financial instruments synergistic? -Comprehensive quantitative research based on content analysis
Source: PLoS One. 2024 Mar 7;19(3):e0298601. doi: 10.1371/journal.pone.0298601 (PMC10919601; doi:10.1371/journal.pone.0298601)
Supplement: S1 Data — (ZIP) [file pone.0298601.s001.zip › Data/地级市Z_F工作报告.zip!/╦─┤¿/┼╩╓a╗¿╩╨/└┤╫╘╣1⁄2╓┌║┼í╛┬φ┐╦╩2╛▌═°í┐_2017.docx]

各位代表：

　　现在，我代表市人民政府，向大会报告工作，请予审查，并请政协委员和其他列席人员提出意见。

　　过去五年的工作回顾

　　过去的五年，是极不平凡的五年。面对全球经济持续低迷，国内经济下行压力较大，钢铁、煤炭等行业产能严重过剩等严峻复杂形势，在省委、省政府和市委的坚强领导下，在市人大、市政协的监督和支持下，市政府团结全市各族人民，主动适应经济新常态，攻坚克难、砥砺前行，统筹做好稳增长、促改革、调结构、惠民生、防风险各项工作，经济运行稳中向好，结构调整深入推进，城市品质显著提升，民生福祉持续改善，完成了市九届人大一次会议确定的主要目标任务，开启了攀枝花转型升级、科学发展的新征程。

　　——坚持抢抓机遇，开创了“四区驱动”发展新格局。争取国家发改委发布了《钒钛资源综合利用和产业发展“十二五”规划》，设立了攀西国家战略资源创新开发试验区；推动钒钛产业园区升级为国家钒钛高新技术产业开发区；获批建设国家现代农业示范区；创建中国阳光康养产业发展试验区，首创康养理念，成功举办首届中国康养产业发展论坛，创办国内首家康养学院，“孝敬爸妈，请带到攀枝花”广为传颂。

　　——坚持稳中求进，实现了经济规模和质量效益新提升。综合实力显著增强。九大发展指标实现大幅增长：GDP由645.66亿元增加到1014.68亿元；规模以上工业增加值年均增长11.5%；累计完成全社会固定资产投资2966.62亿元，是前五年的2.25倍；社会消费品零售总额由165.68亿元增加到316.9亿元；城乡居民人均可支配收入分别由19735元、7627元提高到32860元、14057元；非公经济占GDP比重由41.6%提高到49.5%；商事主体由4.91万户增加到7.19万户；金融业总资产由1538亿元增加到2326亿元。五项控制指标明显下降：万元GDP的能耗、二氧化碳排放量和建设用地分别下降33.6%、39%、34.5%，二氧化硫、氮氧化物排放总量分别削减24.7%、34.3%。产业转型升级成效明显。矿业、钒钛、机械制造、新能源、新材料等重点产业加快发展，工业逐步由钢铁经济为主向钒钛、钢铁经济并进转变、由初级产品向精深加工转变。建成全国首个国家级出口芒果质量安全示范区，部级标准化示范园（场）增加到21个，国家在川高效节水示范项目全部落户我市，农业向标准化品牌化国际化发展。实施重大科技项目带动战略，国家级高新技术企业由14家增加到43家，高新技术产业产值由45.5亿元增加到390亿元，科技对经济增长的贡献率达到55%。

　　——坚持城乡统筹，塑造了阳光花城、康养胜地新形象。发展空间有序拓展。加快新区建设，炳二、炳三区基本成型，渡仁片区开发次第展开，“都市环+放射组团”的阳光型城市架构形成，建成区面积由60.9平方公里扩大到86.5平方公里，城镇化率由61%提高到65.34%、居全省第二。基础设施大幅改善。区域综合交通枢纽建设取得重大突破，丽攀高速攀枝花段建成通车，成昆铁路扩能改造、攀大高速开工建设，国省干道改扩建全面完成，北京、重庆、深圳、上海、武汉航线相继开通。城市路网日趋完善，通村公路全部硬化。水利、电力、通信等设施不断完善。小城镇和新村建设成效显著。8个镇被列为全国重点镇，红格获评“中国特色小镇”，建成旅游新村16个、幸福美丽新村108个。生态环境大为改观。实施“五创联动”，国家卫生城市、省级环保模范城市通过复审，创建为省级森林城市、省级园林城市，荣获全国文明城市提名城市、中国阳光康养示范城市等称号，跻身全国十佳养老城市、全国呼吸环境十佳城市，阿署达、普达等康养项目加快推进，“阳光花城·康养胜地”品牌成功打响。

　　——坚持改革开放，取得了南向门户建设新进展。以改革促开放。行政审批项目减少46.3%，非行政许可审批事项全部取消，工矿废弃地复垦利用纳入全国试点，承包地确权颁证基本完成，商事登记改革全面推进，国资国企改革稳步开展，党政机关公务用车制度改革全面完成，在全省率先实现医联体全覆盖。以开放促发展。与川滇黔毗邻市州、成都市、北京市西城区、澳门、台湾及俄罗斯、乌克兰等扩大开放合作，成功举办中国·攀枝花—东盟国际商贸合作交流活动，建成全国首批、全省首个国家外贸转型升级专业型示范基地。

　　——坚持成果共享，增进了人民群众新福祉。民生工程有力推进。完成民生投资188亿元，办成一大批民生实事。扎实推进脱贫攻坚，减贫71489人，贫困发生率从16.8%降至2.5%。解决25.3万农村人口饮水安全问题。农村民居地震安全工程建设正式启动。社会事业协调发展。学前三年毛入园率97.6%，义务教育基本均衡县评估成为继成都之后全省第一个全域通过国检的市州，高考本科上线率八连升，职业教育、地方高校成绩突出。公共文化服务在全省实现“四个率先”，三线建设博物馆建成并升格为国家级博物馆。医疗卫生发展水平区域领先，公立医院全部取消药品加成，全面两孩政策稳步实施。竞技体育和群众体育活动蓬勃开展。社会保障提质扩面。在全省率先实现居民社会养老保险城乡一体，新农合、城镇居民医保、城镇居民养老保险实现制度性全覆盖。在全省率先启动棚户区改造并实施货币化安置。就业形势保持稳定。社会治理有力有序。“六五”普法成效显著，国防动员和后备力量建设有新进步，连续四届荣获“全国双拥模范城”，成为西南三省唯一的全国和谐社区建设示范城市，群众安全感测评满意率稳居全省前列。

　　——坚持改进作风，展现了政府建设新气象。扎实开展党的群众路线教育实践活动、“三严三实”专题教育、“两学一做”学习教育，认真贯彻落实中央八项规定和省、市十项规定，建立健全纠风工作机制，查办群众身边“四风”和腐败问题案件301件，党风廉政建设社会评价和政风行风群众满意度持续提升。狠抓法治政府建设，强化审计监督，政府重大行政决策全部纳入合法性、廉洁性审查，行政权力事项基本实现全业务全流程网上公开运行。被评为全省法治宣传教育先进城市。

　　民族、宗教、人防、国安、气象、供销、档案、邮政管理、外事侨务、地方志、关心下一代、妇女儿童、残疾人、对口援助援建等工作都取得新成绩。

　　各位代表，刚刚过去的2016年，全市上下凝心聚力、苦干实干，完成了GDP过千亿目标，财政收入增速扭负为正，多项经济指标在全省位次前移，实现了“十三五”良好开局。地区生产总值增长8%，规模以上工业增加值增长9%，全社会固定资产投资增长8%，社会消费品零售总额增长10.7%，地方一般公共预算收入增长6.4%，城乡居民人均可支配收入分别增长8.2%、9.3%。

　　过去一年，市政府重点抓了六个方面的工作。一是强化调度保目标，发展态势稳中向好。对经济运行实行“一月一调度”，及时解决工作中的困难和问题，推动经济一步一步爬坡。新增规模以上工业企业18户，投资、财政收入等下行指标止滑回升。二是分类施策促改革，“三去一降一补”扎实落地。淘汰钢铁落后产能150万吨，关闭小煤矿10处、退出产能114万吨；化解房地产库存120万平方米；设立10亿元兴攀产业发展基金和20亿元PPP项目财政支持基金，为“红名单”企业提供应急转贷资金4.75亿元；取消行政事业性收费42项，完成“营改增”扩面，实现资源税从价计征，减轻企业税费负担9.78亿元，为企业争取电费优惠7.94亿元、降低铁路运费3.46亿元；高炉渣提钛等产业化关键技术取得重大突破。三是突出重点抓项目，发展后劲逐步增强。抓实“项目推进年”工作，全年新签约项目373个、协议资金751.35亿元，到位资金568.22亿元、增长4.1%，新开工项目305个、总投资307.16亿元，完成全社会固定资产投资696.31亿元。四是精准发力补短板，脱贫攻坚首战告捷。整合各类扶贫资金9.9亿元，实施扶贫专项方案10个、年度扶贫专项工作计划15个，1057人易地搬迁安置、20个贫困村退出、6457人脱贫的任务圆满完成。五是建管并重强功能，城市品质不断提升。天星湖、市政务中心、火车南站等开工建设，国家园林城市、森林城市创建指标全部达标，环境空气质量优良率首次达到100%、居全省第一，荣获2016中国智慧城市创新奖。六是顺应民意办实事，社会大局保持稳定。19件民生大事、144件民生事项全面完成。养老金按时足额发放，城镇登记失业率4.01%。保障性安居工程目标任务超额完成。全面取消二滩网箱养鱼，库区水质向好。科学应对“9·19”特大暴雨等自然灾害，灾后重建有序推进。有效处置央视曝光的环境污染问题，依法关停整顿企业37家。

　　各位代表，过去五年的成绩来之不易。这是省委、省政府和市委坚强领导的结果，是全市上下群策群力、拼搏实干的结果。在此，我代表市政府，向全市各族人民，向人大代表、政协委员、各民主党派、工商联、无党派人士、各人民团体，向驻攀解放军指战员、武警官兵、政法干警和民兵预备役人员，向各位老领导、老同志，表示衷心的感谢！

　　五年的实践充分证明：市委、市政府抢抓机遇、深化改革、创新发展，大力实施“四个加快建设”、不断深化“四区驱动”的战略和举措是完全正确的，只要我们坚持一张蓝图绘到底，一茬接着一茬干，就一定能使蓝图变实景。五年的实践告诉我们：攀枝花是一片充满希望和活力的热土，攀枝花人民富有创造力，攀枝花干部想干事、能干事、干成事，只要我们进一步解放思想、勇于担当，坚定不移转方式、调结构、促改革、补短板，就一定能打赢转型升级攻坚战。五年的实践启示我们：在新的起点上推进攀枝花发展取得新成就，必须用好比较优势，做好钒钛、阳光两篇文章，把资源优势充分转化为产业优势和经济优势。必须坚持科学发展，遵循经济、自然、社会三大规律，实现更高质量的发展。一要遵循经济规律，坚持用市场经济的理念思考问题，用市场机制和市场化手段破解发展中的难题，处理好政府与市场的关系，增强驾驭市场经济的能力；二要遵循自然规律，坚持走生态优先、绿色发展之路，处理好经济发展与生态环境保护的关系，保护利用好青山绿水、蓝天白云、阳光花城这一最宝贵的财富和最大的优势；三要遵循社会规律，坚持以人民为中心，处理好经济发展与社会建设的关系，打造更加公平正义的社会环境。这些经验和启示，我们倍加珍惜。

　　同时，我们也清醒地看到，在前进道路上还有不少困难和问题，工作中还存在缺点和不足。经济总量小、产业结构不合理、资源环境约束趋紧、系统抗风险能力弱的基本市情没有根本改变；改革创新、科技支撑能力仍然薄弱，重大投资项目不多，民间投资意愿不足；交通仍然是制约发展的突出瓶颈，城市基础设施薄弱，公共服务能力偏低，城乡区域间发展不协调的问题依然突出，城市低收入群体和一些边远山区群众生产生活还存在不少困难。部分政府工作人员的能力、作风、执行力与工作要求不相适应；用好用足用活政策还有很大空间。我们将正视困难和问题，采取切实措施，认真加以解决。

　　今后五年的目标任务

　　今后五年，是攀枝花加快产业结构调整、转型发展的关键时期，也是在全省高水平率先全面建成小康社会的决胜期。我们要始终保持专注发展、转型发展战略定力，在贯彻落实市第十次党代会精神上凝神聚焦发力，以踏石留印、抓铁有痕的精神，把党代会确定的目标任务细化、实化、具体化，奋力推动攀枝花在全省高水平率先全面建成小康社会。

　　今后五年政府工作的总体要求是：高举中国特色社会主义伟大旗帜，以邓小平理论、“三个代表”重要思想和科学发展观为指导，深入学习贯彻党的十八大、十九大精神和习近平总书记系列重要讲话精神，紧紧围绕“四个全面”战略布局，牢固树立新发展理念，坚持稳中求进工作总基调，以提高经济发展质量和效益为中心，以推进供给侧结构性改革为主线，以创新为动力，突出绿色发展，大力推进中国钒钛之都、阳光花城、康养胜地和四川南向门户“四个加快建设”，不断深化攀西国家战略资源创新开发试验区、国家现代农业示范区、国家钒钛高新技术产业开发区、中国阳光康养产业发展试验区“四区驱动”，加快形成四川新的经济增长点，在全省高水平率先全面建成小康社会。

　　今后五年全市经济社会发展的主要目标是：地区生产总值增速高于全省，2019年在全省高水平率先全面建成小康社会，2021年建成区域性现代化中心城市。

　　——产业结构更加优化。着力推进供给侧结构性改革，深入推进“三去一降一补”，一产做精、二产做强、三产做大，阳光康养、特色农业成为全国标杆，钒钛钢铁、机械制造、新材料、新能源等产业迈上中高端水平，高质量建成国家现代农业示范区、世界级钒钛产业基地、中国阳光康养产业发展试验区。

　　——生态环境更加优美。长江上游生态屏障建设取得明显成效，彰显阳光花城特色的生态景观加速形成，森林覆盖率提高到60.3%以上，建成区绿化覆盖率提高到42%以上，人均公园绿地面积提高到14平方米以上，环境空气质量优良率保持全省领先，地表水质量100%达到或好于Ⅲ类水体，土壤环境质量总体稳定，力争全面实现“五创联动”创建目标。

　　——人民生活更加幸福。城乡居民收入保持全省领先并超过全国平均水平，城乡基本公共服务均等化基本实现，就业更加充分，教育更加公平优质，医疗卫生服务更加高效，交通出行更加便捷，社会保障更加完善，社会治理更加有序，全市人民获得感、幸福感、自豪感和归属感大幅提升。

　　根据上述总体要求和奋斗目标，今后的主要任务是：

　　一、突出创新驱动，壮大中国钒钛之都实力

　　围绕攀西国家战略资源创新开发试验区建设，大力实施省委“一号工程”，加快建成以“五个一工程”为支撑的世界级钒钛产业基地。

　　全面创新改革，政策先行先试。争取一批支持攀西国家战略资源创新开发试验区建设的试点政策，设立钒钛产业发展基金，开展采矿用地改革和电冶联营等试点，推进红格南矿区开发，构建加快钒钛资源创新开发的政策支撑体系。促进攀钢、钢城等大企业改革。

　　坚持科技兴市，突破关键瓶颈。建好钒钛科技孵化器、石墨产业研究中心等科研平台。推动国家层面建立重大科研项目协调推进机制，持续推进重大科技攻关项目全球招标，突破关键核心技术。加快高炉渣提钛、钒电池、钛及钛合金深加工、碳基材料等重大科技攻关项目的产业化进程。推进军民融合，使更多钒钛、钢铁产品进入军工领域。加快国家知识产权示范城市建设。

　　着眼高点定位，打造“五个一工程”。建实一个“高”“新”特色鲜明的国家钒钛高新区，建设一个聚集全国钒钛交易资源的国家钒钛交易所，建强一个部省市共建、产学研协同的国家钒钛重点实验室，建成一个具有标准主导权的国家钒钛质检中心，建好一批有实力的钒钛企业。

　　强化机制建设，建设创新创业型社会。大力开展大众创业、万众创新。积极推进国家创新型城市试点，建立企业主导的产学研协同创新机制，推动研发平台布局向企业倾斜，培育国家、省级企业技术中心5家以上。健全创新创业服务体系，创建科技和金融结合试点城市。实施人才强市战略，建立更具竞争力的人才引进和激励机制。

　　二、突出绿色主题，提升中国阳光花城品质

　　统筹规划、建设、管理三大环节，优化生产、生活、生态三大空间，实现城市功能、特色景观、对外形象的全面提升。

　　推进城市扩容提质。以全域规划、多规合一为引领，健全市域空间管制、综合交通体系、绿地系统布局等规划，构建现代新型城镇体系。以城市骨架和市域路网建设为依托，完善地下管网等基础设施，高标准推进新区建设，有序拓展城市空间，建成区面积扩大到100平方公里以上。加快旧城改造和老工业基地改造，力争成为“先生产、后生活”人居环境提升改造全国试点城市。以城市设计和精细化管理为抓手，构建“适树适花、集群成景、特色鲜明”的生态景观体系，提升城市形象。

　　打造绿色转型发展示范城市。坚定不移推进绿色转型发展，加快培育钒钛、石墨、清洁能源为代表的新兴产业。大力推行清洁生产，建设绿色工厂、绿色园区、绿色矿山，力争万元工业增加值能耗、水耗分别下降25%、23%以上。深化资源综合利用，固体废弃物综合利用率提高到75%。严把环境准入关口，不符合环保要求的项目一律不上，坚决不走“先污染后治理”的老路。

　　巩固提升生态环境质量。牢固树立“绿水青山就是金山银山”的意识，强力推进生态建设和环境保护。开展大规模绿化攀枝花行动，实施主城区提质增绿、金沙江干热河谷生态脆弱区综合治理等工程。打好大气、水、土壤污染防治“三大战役”，确保工矿企业污染排放问题基本解决。保护和利用好水电开发形成的五大湖区。

　　三、突出融合发展，丰富中国康养胜地内涵

　　大力发展“康养+”，构建中国阳光康养产业发展试验区产业体系，制定阳光康养标准，引导康养产业快速发展。

　　大力发展“康养+农业”。深入推进国家现代农业示范区建设，突出高品质、名优特，升级农产品质量，增加绿色优质农产品供给，“三品一标”认证超过150个。推进产业化、标准化发展，打造攀枝花芒果等区域品牌；推动农业向休闲观光、农事体验等多功能拓展，形成特色农业和康养产业融合互动局面。

　　大力发展“康养+工业”。引导钢铁、钒钛、机械加工制造业向康养制造延伸，推动医疗、康复辅助器具产业化，发展高档体育器材、高端医疗器械等钒钛终端产品。支持攀钢、攀煤、十九冶、钢城等大企业发展康养产业。

　　大力发展“康养+医疗”。建设全国医养结合试点城市，促进养老与医疗机构双向合作，制定康复护理质量标准，加强康复护理人才培养，构建多层次的康复护理和养老体系，发展现代中医药产业，培育壮大集医疗、康复、保健、养生、养老等为一体的康养医疗产业。

　　大力发展“康养+旅游”。充分发挥“六度禀赋”，全力打造全国阳光康养旅游目的地。创建国家全域旅游示范区，打造一批景观景点，办好欢乐阳光节等品牌活动，发展四季旅游。健全公共交通、汽车营地等公共服务体系，建成国家旅游休闲示范城市。拓展国内市场，开辟国际市场，旅游收入年均增长15%以上。

　　大力发展“康养+运动”。培育户外体育运动产业，打造1—2项有国际影响力的体育活动，争取举办国内外大中型体育赛事。充分发挥现有体育场馆民专两用作用，全面实现全民健身“三纳入”，构建15分钟城市社区健身活动圈。

　　四、突出开放引领，激发四川南向门户活力

　　注重在扩大开放中聚集要素、拓展空间、赢得优势，全面提升开放合作层次和城市发展活力。

　　加快通道建设。全面打响交通建设“五年大会战”，力促成昆铁路扩能改造、丽攀高速、攀大（四川境）高速和攀宜高速建成通车，开工建设攀大铁路、攀昭遵铁路、攀盐（源）高速公路，形成“一环六射”高速路网，打造1小时经济圈；加快机场改造升级，增加航班航线数量，增开国内主要大城市航班；研究对接昆明—永仁城际铁路项目，大力争取高铁项目、攀枝花港和金沙江攀枝花—水富段航道建设；构建完善轨道交通、管道运输、主次道路便捷衔接的城市交通网络，基本建成区域性综合交通枢纽。

　　加大开放力度。主动融入国家“一带一路”和长江经济带建设，面向长三角、珠三角、港澳台等重点区域加强资本引进、市场对接。深化与成都市、北京市西城区的友好合作机制，强化央企项目对接，引入首都科技创新、教育医疗等资源。加强与东盟十国交流合作，持续办好攀枝花—东盟国际商贸交流活动，建立东盟跨境电子商务园，建成省级对外开放合作示范平台。与凉山共建攀西经济区、攀西城市群，借力成德绵全面创新改革政策和军民融合发展机制，深化与川滇黔毗邻市州的区域合作。用好攀西国家战略资源创新开发试验区的政策优势，推动设立海关特殊监管区域，面向欧盟、俄罗斯、乌克兰等引智、引技。

　　五、突出富民惠民，高水平率先全面建成小康社会

　　坚持把保障和改善民生作为一切工作的出发点和落脚点，办好民生实事，深化社会治理，不断增强人民群众获得感，提升人民群众的幸福感、自豪感和归属感。

　　全面打赢脱贫攻坚战。聚焦“两不愁、三保障”和“四个好”目标，全面落实“六个精准”要求，整合资源，加大投入，大力实施“六大扶贫工程”，确保2018年现行贫困标准线下的贫困人口、贫困村全部脱贫退出。

　　大力发展民生事业。加大民生投入，不断提升公共服务质量和水平。把教育放在优先发展的战略地位，扩大公办学前教育资源，提升农村义务教育质量，做精做强两类高中教育，组建综合性职业技术学院，高水平建设地方特色大学，办好攀枝花国际康养学院，加快建成区域教育高地。坚持文化惠民，深化公共文化服务体系建设，打造一批有竞争力的文化产品和企业，努力建成区域文化高地。打好医药卫生体制改革攻坚战，建好花城新区医院、市妇女儿童医院，力争成为全国健康城市示范市，全面建成区域医疗卫生高地。强化防灾减灾救灾能力建设，大力实施农村民居地震安全工程，全面完成危旧房棚户区和农村危房改造。办好就业促进、社会保障、交通出行等民生实事。

　　切实提高社会治理水平。大力推进法治攀枝花、平安攀枝花、和谐攀枝花建设。建立政府主导、社会参与、多元供给的社会服务模式，推动社会治理向激励引导型转变。建立完善立体化信息化社会治安防控体系，创建全国公共安全视频监控建设联网应用工程示范城市，完善社会矛盾纠纷排查预警和调处化解综合机制，开展“七五”普法，深化和谐社区建设。落实“四个最严”要求，争创国家食品安全城市。强化安全生产“党政同责、一岗双责、齐抓共管、失职追责”制度，推动安全形势持续向好。深入开展双拥共建活动，加大国防动员后备力量建设。建成国家新型智慧城市。

　　六、突出责任担当，打造为民务实清廉政府

　　推进依法行政。坚持法治思维和法治方式，形成办事依法、遇事找法、解决问题用法、化解矛盾靠法的法治良序。严格执行权力清单和责任清单，按照法定权限和程序履行职责。严格依法决策，强化综合执法，加强执法监督。全面推进政务公开，自觉接受党内监督、人大监督、民主监督、司法监督、审计监督、社会监督和舆论监督，认真办理人大代表建议和政协提案。

　　提升执行力。“一分部署，九分落实”，坚持高标准、严要求、重实效，以雷厉风行的作风和钉钉子精神抓落实。强化目标导向和问题导向，对标先进立目标，对着问题开药方。完善月度经济运行调度工作机制，推行清单制和责任制，对重点工作落实责任、倒排工期、挂图作战、台账式管理，一项项工作推进，一个个问题解决。深化行政体制改革，清理重复交叉职能。强化绩效管理，严格问责不作为、乱作为、推诿扯皮、效能低下的人和事。

　　保持清正廉洁。坚定不移深入推进全面从严治党，严格落实“一岗双责”，坚持把纪律和规矩挺在前面，深入推进党风廉政建设和反腐败斗争，严格执行中央八项规定精神和省委省政府十项规定，坚决防止“四风”问题反弹。深化源头防腐，严格落实谈话函询、民主生活会、述职述廉等制度。强化行政监察，严肃查办各类违纪违法案件，保持惩治腐败高压态势，营造干部清正、政府清廉、政治清明的政务环境和发展环境。

　　2017年的重点工作

　　2017年是实施“十三五”规划的重要之年，也是推进供给侧结构性改革的深化之年。做好2017年工作，责任重大。全市经济社会发展的主要预期目标是：地区生产总值增长7.5%以上；全社会固定资产投资增长7%以上；地方一般公共预算收入增长4%以上；社会消费品零售总额增长10%以上；城乡居民人均可支配收入分别增长8%、9%以上；城镇登记失业率控制在4.5%以内；居民消费价格指数涨幅控制在3.5%以内；节能减排完成省下达任务。

　　围绕上述目标，重点做好六个方面的工作：

　　一、着力稳定经济增长，巩固稳中向好发展态势

　　把抓项目、抓政策、抓资金“三抓”作为经济工作的主抓手。

　　抓项目扩投资。抓好“项目年”工作，坚定不移大抓项目、抓大项目、抓好项目。改进投促方式，压实“领导带队、部门紧促、县（区）紧盯”责任，力争签约一批5亿元以上的龙头项目。督导签约项目落地，实现到位资金580亿元以上。推行“一个项目、一名领导、一套班子、一个方案、一抓到底”的项目推进机制，健全项目审批绿色通道，做好土地、水、电、路等要素保障，开展闲置土地专项清理，实施5亿元以上重大项目22个，完成全社会固定资产投资745亿元以上。

　　抓政策谋红利。在用好用足用活政策上下功夫，围绕财税金融、供地用地、直供电、资源配置管理、生态补偿等五大领域，着力探索攀西战略资源创新开发试验区先行先试政策。衔接长江经济带生态保护补偿政策，推动建立金沙江流域水环境生态保护补偿机制。嫁接成德绵全面创新改革政策，争取科技成果混合所有制权属改革试点。出台支持民营经济发展的政策措施，建立完善政府领导联系行业协（商）会制度。帮助盐边县、米易县、仁和区争取资源补偿等民族地区政策。

　　抓资金强保障。积极争取中央和省级财政资金，出台考核激励办法，纳入目标管理。用好PPP政策，力争更多项目由社会资本建设。深化政银企合作，创新融资模式，创建省级科技和金融结合试点城市。用好兴攀产业基金，设立钒钛产业发展基金和康养产业发展基金。改革公共资金使用方式，强化事前评估和事中监管。出台债券融资工作方案，推动企业上市融资。

　　二、着力深化创新改革，推动攀西国家战略资源创新开发试验区建设取得重大突破

　　力推改革措施落地。深入推进“三去一降一补”，关闭7处小煤矿，东区、西区、仁和区棚改原则上全部实行货币化安置，探索开展市场化、法治化债转股，帮助企业降本增效。抓好“放管服”、国资国企、审计、“五证合一”、供销社等改革。推进“两保合一”，建立统一的城乡居民医疗保险制度。着力先行先试，推动钒钛磁铁矿露天采矿场、排（弃）土场、尾矿库等用地纳入采矿用地改革试点；编制电冶联营试点方案，筹建地方售电公司；争取减征或即征即退钒钛产业及其废弃物利用的增值税，税款优惠用于企业新技术研发；启动红格南矿区开发前期研究工作。

　　实施“五个一工程”。支持钒钛高新区构建“一区多园”空间布局，建立“国家千人计划”工作站和军民两用技术转移孵化中心，推动高炉渣提钛及氯化法钛白、钛合金无缝管等项目加快建设；支持钒钛交易所建立会员推广机制，力争年交易量增加80亿元；支持国家钒钛重点实验室、四川钒钛材料工程技术研究中心完善硬件平台，改革运行体制；支持国家钒钛质检中心筹建全国钒钛标准化技术委员会，完成可行性报告和筹建方案。

　　健全“双创”支撑平台。加快建设创新创业孵化园，完善科创通攀枝花分平台和四川钒钛产业技术交易平台，打造国家级创业孵化链条建设示范单位，建设清华启迪之星攀枝花基地、软通动力攀枝花创新综合体，与复旦大学开展“双基地模式”合作，培育2家省级众创空间。

　　三、着力发挥比较优势，加速产业转型升级步伐

　　推动农业提质增效。推进农业供给侧结构性改革，狠抓标准化生产和品牌创建。启动有机产品认证示范区建设，建成农产品质量安全追溯平台。打造攀枝花芒果品牌，启动工矿废弃地复垦种植芒果试点。实施新型农业经营主体培育示范工程，建成3—4个现代农业集成创新示范农庄。加强耕地保护，深化农村土地制度改革，建成市级农村土地产权交易平台。加快推进“十三五”重点水利建设项目，建成马鞍山水库等水利枢纽工程。做好第三次全国农业普查工作。第一产业增加值增长3.8%以上。

　　推动工业升级提效。坚持工业强市，培育2—5家国家高新技术企业，推动12家企业升规。落实《中国制造2025》，打造钒钛低（微）合金钢机械深加工等新材料基地。着力振兴实体经济，支持攀钢、钢城等多元发展；抓好煤炭生产、调运，用好优惠电；加快处置“僵尸企业”，推进兼并重组；大力推动军民融合，促成与成飞集团、中船重工等大型军工企业的合作。规模以上工业增加值增长8.5%以上。

　　推动服务业加快发展。完成康养产业规划编制，打造标准化康养基地，发展康养地产，做实“康养+”。启动国家全域旅游示范区创建工作。出台促进消费增长措施，新增20户限额以上商贸流通企业。打造攀枝花—南亚物流基地，与宜宾开展铁水联运，加快建设省级现代物流示范城市。引进更多第三方知名电商平台，拓展攀枝花产品的网购网销渠道。加大外贸企业培育力度。第三产业增加值增长8%以上。

　　四、着力加强环境保护，筑牢生态安全屏障

　　全面打响污染防治“三大战役”。坚决整治大气、水、土壤污染问题，特别要加强工矿企业污染排放、矿区土壤重金属和农村面源污染整治防治，让天更蓝、山更绿、水更清。实施工业污染源全面达标排放计划，坚决关停整改不达标企业；加强环境监管，严控城市扬尘、噪音和餐饮油烟污染；建立健全环境改善长效机制，确保环境空气质量优良率全省领先。

　　持续推进长江上游生态屏障建设。严格划定生态保护红线，深入实施天保工程，大力推进矿山迹地治理、道路及水系生态廊道建设，创建金沙江干热河谷区生态恢复与治理示范区，力争建成国家森林城市。完成湿地资源保护与利用中长期规划编制，申报建设湿地公园。积极推行河长制，加大饮用水和良好水体保护力度。

　　五、着力加大统筹力度，促进城乡区域协调发展

　　提升城乡功能品质。修编完善城市总规和控制性详规。加快推进火车南站、天星湖、城市轨道交通、东区后山公路、观音岩引水工程等项目建设。结合国有企业“三供一业”分离移交，加快推进老旧小区改造提升工程，整治30个老旧小区的居住环境。加大集镇建设力度，培育一批特色小镇，新建40个幸福美丽新村，创建30个省级“四好村”。建立大数据中心，整体推进智慧城市建设。推行城区网格化管理，启动城市管理综合执法试点。

　　激发县域经济活力。指导县（区）调整优化发展定位，培育各具特色的“板块经济”。支持东区加快发展现代服务业、打造区域性现代化中心城区，西区加快培育支柱产业、打造南向开放先行区，仁和区加快发展战略性新兴产业、打造城乡一体化示范区，米易县全域发展特色精品农业、打造阳光时尚花园城，盐边县加快北部跨越南部提升、打造绿色崛起样板县。

　　六、着力增进民生福祉，推动社会事业全面进步

　　办好民生实事。深入实施民生工程，全面完成省级民生任务，办好10件市级民生实事：完成213户“9·19”特大暴雨受灾住房重建；新建（硬化）50公里乡村便民道路，硬化13公里民族地区道路；新增30个行政村通有线光纤网络；建成市芒果批发市场；完成市食品安全检测中心主体工程建设；完成市残疾人康复托养中心主体工程建设；完成市生活垃圾焚烧发电项目主体工程建设；建成投用成都外国语学校攀枝花分校；投放53台新能源公交车，新建855个公共停车位；完成花城新区独松梁子生态修复治理工程。

　　打好脱贫攻坚战。把脱贫攻坚作为头等大事，扎实推进“六大扶贫工程”，抓实农村危房改造、异地扶贫搬迁、彝家新寨建设、扶贫小额贷款等重点脱贫项目，加快完善贫困地区基础设施和公共服务设施，拓展增收致富渠道。确保35个贫困村退出、8697人脱贫。

　　守好社会稳定、安全生产、食品药品安全、生态环境“四条底线”。加强社会治安综合治理，健全群众诉求表达、利益协调和权益保护机制，及时排查化解社会矛盾。完善应急管理体系，强化地震、气象、水情监测预报预警，治理17个地质灾害隐患点。建立融资风险分担机制，确保不发生系统性、区域性金融风险。深化“打非治违”和安全隐患排查治理，积极推进小煤矿关闭工作，特别是要加强对矿山、尾矿库等重点领域的排查整治，依法打击安全生产违法违规行为，坚决遏制重特大安全事故发生。强化食品药品安全监管，实行最严格的环境保护制度，让人民群众吃上放心的食品、呼吸上新鲜的空气、喝上干净的水。

　　各位代表！回顾过去，成绩催人奋进；面向未来，倍感责任重大、使命光荣。让我们紧密团结在以习近平同志为核心的党中央周围，在省委、省政府和市委的坚强领导下，坚定信心、保持定力，解放思想、改革创新，勇于担当、苦干实干，以优异成绩迎接党的十九大和省第十一次党代会胜利召开！
